# Inside the Joint of Inflammatory Arthritis Patients: Handling and Processing of Synovial Tissue Biopsies for High Throughput Analysis

**DOI:** 10.3389/fmed.2022.830998

**Published:** 2022-03-14

**Authors:** Achilleas Floudas, Aine Gorman, Nuno Neto, Michael G. Monaghan, Zoe Elliott, Ursula Fearon, Viviana Marzaioli

**Affiliations:** ^1^Molecular Rheumatology, Trinity Biomedical Sciences Institute, Trinity College Dublin, Dublin, Ireland; ^2^European League Against Rheumatism (EULAR) Centre of Excellence, Centre for Arthritis and Rheumatic Diseases, St. Vincent's University Hospital, University College Dublin (UCD), Dublin, Ireland; ^3^Trinity Centre for Biomedical Engineering, Trinity Biomedical Sciences Institute, Trinity College Dublin, Dublin, Ireland

**Keywords:** inflammatory arthritis, synovial membrane, functional analysis, single cell analysis, synovial biopsies, new technologies

## Abstract

Inflammatory arthritis is a chronic systemic autoimmune disease of unknown etiology, which affects the joints. If untreated, these diseases can have a detrimental effect on the patient's quality of life, leading to disabilities, and therefore, exhibit a significant socioeconomic impact and burden. While studies of immune cell populations in arthritis patient's peripheral blood have been informative regarding potential immune cell dysfunction and possible patient stratification, there are considerable limitations in identifying the early events that lead to synovial inflammation. The joint, as the site of inflammation and the local microenvironment, exhibit unique characteristics that contribute to disease pathogenesis. Understanding the contribution of immune and stromal cell interactions within the inflamed joint has been met with several technical challenges. Additionally, the limited availability of synovial tissue biopsies is a key incentive for the utilization of high-throughput techniques in order to maximize information gain. This review aims to provide an overview of key methods and novel techniques that are used in the handling, processing and analysis of synovial tissue biopsies and the potential synergy between these techniques. Herein, we describe the utilization of high dimensionality flow cytometric analysis, single cell RNA sequencing, *ex vivo* functional assays and non-intrusive metabolic characterization of synovial cells on a single cell level based on fluorescent lifetime imaging microscopy. Additionally, we recommend important points of consideration regarding the effect of different storage and handling techniques on downstream analysis of synovial tissue samples. The introduction of new powerful techniques in the study of synovial tissue inflammation, brings new challenges but importantly, significant opportunities. Implementation of novel approaches will accelerate our path toward understanding of the mechanisms involved in the pathogenesis of inflammatory arthritis and lead to the identification of new avenues of therapeutic intervention.

## Introduction

Inflammatory Arthritis (IA) including Rheumatoid Arthritis (RA) and Psoriatic Arthritis (PsA) are important chronic Rheumatic and Musculoskeletal Diseases (RMD) worldwide causing significant joint destruction, disability, increased mortality and are associated with co-morbidities (1–3). The direct and indirect cost of inflammatory arthritis is significant for the patient, their families and society at large. Importantly, recent studies highlight that the burden associated with RA and PsA is underreported and is significantly higher than previously calculated while incidence rates are increasing globally (4, 5). Current advances in clinical practice and the increasing use of ultrasonographic and arthroscopic technologies and patient partnerships have led to increased availability of synovial biopsies (6, 7). This has fuelled recent advances in understanding the underlying immunological mechanisms involved in synovial inflammation and the development of targeted biologic therapies which have significantly improved outcomes for patients with IA. However, responses may be sub-optimal or associated with adverse events, there are no cures, and therefore patients require lifelong treatment. Indeed a substantial percentage of patients still do not achieve low disease activity or remission, with studies showing that only 18% of RA patients reach a state of low disease activity on their first treatment, while 10% of patients are refractory to multiple treatments (8). Another study examining both RA and PsA, demonstrated remission rates on biologic disease-modifying antirheumatic drug (bDMARD) therapy were higher in PsA compared to RA, at 1 and 12 years following biologic therapy (9). The low number of patients achieving lasting remission is indicative of the heterogeneity of IA and the potential for disease endotypes with common clinical manifestations but differential immune mechanism involvement (10). Early intervention when radiographic damage is low is a key predictor of sustained DMARD free remission (11). Therefore, there is an urgent and unmet need to identify individuals at risk, biomarkers of disease and response to treatment in order to achieve patient stratification and apply the right treatment early in disease. In addition to effective patient stratification characterization of cell-cell interactions at the site of inflammation, the synovium, is required for the successful development of novel targeted therapeutic interventions.

## Synovium Histological Analysis and Response to Therapy: Contributions and Limitation

To date, patient' stratification and disease pathotype stratification, as well as response to therapy has been mainly obtained with histological analysis of the synovium. Although RA and PsA have many common clinical manifestations, we and others have demonstrated significant differences in the vascular pattern, immune-cell infiltrates and the invasive lining-layer at the site of inflammation (2–16), which may be associated with the distinct pattern of joint involvement and bone erosion observed between RA and PsA (12–16). Angiogenesis is dysregulated in both conditions, with the formation of elongated, torturous blood vessels, a distinct phenotype in the PsA joint (17). In contrast, lining layer hyperplasia is more striking in RA than in PsA (18). Findings vary in the histological analysis of PsA and RA, with studies displaying extensive infiltration of polymorphonuclear cells associated with PsA (14), and increased frequency of macrophages, T-cell and B-cell subsets associated with RA, while some studies show comparable frequencies in both conditions (14, 19, 20). The synovial infiltration of immune cells including T cells, B cells, plasma cells, monocytes, neutrophils, NK cells and potentially innate lymphoid cells together contribute to aberrant inflammation, ultimately resulting in bone erosion, cartilage destruction and loss of function of the joint (21–24). The presence of B cell and T cell rich lymphoid aggregates has been linked to more aggressive and erosive RA disease (25). These ectopic lymphoid structures could enable altered B and T cell activation and effector function resulting in potentiation of inflammation (6, 26). CD68 macrophage accumulation in the synovial sub-lining layer and perivascular mononuclear infiltration were found to be prominent in RA tissue, especially in clinically-involved joints (27). Indeed, sub-lining expression of CD68 is the only cell marker to date that correlates with response to therapy, regardless of therapeutic intervention (28, 29). Pontifex et al., have shown in PsA that both CD3 and CD68 in the synovium decrease in response to anti-IL1 and anti-TNF therapy, with CD3 expression correlating with disease activity (30); in addition, in a multi-center study focussing on patients pre/post rituximab treatment, CD68 has been shown to correlate with disease activity (29). Patients taking prednisolone presented a reduction in synovial macrophages CD68, CD4 and CD5 (T and B cells) and CD38 (plasma cells) and CD55 [fibroblast-like synoviocyte (FLS)] cells post-treatment (6, 31). Histological analysis has also been used as a tool to distinguish between disease pathotypes, with Kruithof et al., underlining that PsA synovium is significantly different from RA in terms of lining layer hyperplasia and PMC infiltration, whereas it presents similarity with SpA synovium (18). In addition, Alivernini et al., suggest histological analysis of ST might be considered an additional tool to discriminate between the two diseases, as observed by the differential distribution of CD117^+^ and CD138^+^ cells among PsA and Ab^neg^ RA (32). As described above, another key difference between RA and PsA is the pattern of neo-vascularization, which has been associated, at the microscopic level, with increased vascularity present in PsA synovium as indicated by the increased number of blood vessels/high power field (33). In addition, Murray-Brown et al., proposed immunohistochemistry as a co-adjuvant tool for therapy selection in a case study of seronegative polyarthritis (34). Stratification of RA patients based on histological characterization of synovial immune infiltrates has resulted in the identification of three potentially distinct endotypes of RA that include the pauci-immune, diffuse-myeloid and lympho-myeloid endotypes (35). RA disease endotype distinction has also been suggested on the basis of differential autoantibody involvement with distinct synovial T cell cytokine responses and worst prognosis in patients positive for anti-citrullinated protein antibodies (ACPA) compared to ACPA negative RA patients (22). Furthermore, synovial B cell infiltrates and lymphoid aggregates are significantly higher in ACPA^+^ RA patients, specifically in those who were naive to treatment (36).

Histological analysis of synovial tissue has also been used to evaluate and predict specific responses to therapy. Rooney at al., showed that histological analysis could be a useful tool for discriminating responder vs. non-responder based on CD3 T cell infiltration (37); similarly, LT-α and TNF-α were observed to decrease in patients responding to Etanercept therapy (38), and B cells following Rituximab therapy (39). RA patients presenting synovial lymphocyte aggregates have been shown to respond better to infliximab therapy, and to be sensitive to TNFi treatment (40). Immunohistochemistry staining for cellular infiltrate, including CD3 and CD68, in a 12 months follow-up study on a patient failing MTX, showed that intra-articular infliximab injection led to a significant decrease over-time of cellular infiltration and pro-inflammatory cytokines (41). In contrast, CD68 macrophage infiltration was not susceptible to JAK/STAT inhibition by tofacitinib, however decreased MMPs and IFN-regulated gene expression in the synovium of RA patients were observed (42). Importantly, recent advances in immunofluorescence analysis with the implementation of novel antibody multiplexing techniques utilizing DNA barcoded antibodies allowing for target co-detection by indexing (CODEX) have the potential to significantly increase the number and resolution of co-detected targets in histological analysis of synovial tissue biopsies (43).

In addition to histological and conventional gene expression analysis of synovial tissue pre/post therapy, *ex vivo* whole tissue synovial explant cultures have also been utilized for pre-clinical proof of concept studies to examine potential regulators of synovial inflammatory responses. While there is extensive literature on the regulation and blockade of pro-inflammatory mediators in primary synovial tissue/fluid cells (synovial fibroblasts, T cells, B cells, macrophages) and in peripheral blood immune cell populations, very few studies have examined the complex multicellular microenvironment of the inflamed joint. Synovial explant cultures spontaneously release pro-inflammatory mediators and maintain the synovial architecture and cell-cell contact of the synovium. Therefore, they are a more patho-physiologically relevant *ex-vivo* model that closely reflect the *in vivo* microenvironment of the inflamed joint. Indeed, the first studies to identify TNFα as a key cytokine involved in driving the inflammatory response in RA utilized RA synovial tissue cultures (44, 45). This led to the development of clinical trials with anti-TNF therapies in RA and later PsA. Subsequent studies utilizing these *ex vivo* models have shown that spontaneous release of pro-inflammatory mediators correlates with clinical disease activity and response to therapy (46, 47), have identified regulators of inflammation (48–50), in addition to examining the effect of therapeutic intervention (51–53). Furthermore, explant conditioned media (ECM) cultured with specific immune cell populations have been utilized to show that the environment of the inflamed joint can induce pathogenic phenotypes in healthy immune cells, thus another potential physiologically relevant model to examine immune cell responses (54).

While these studies are important, they are still limited with regard to in-depth analysis of immune cell involvement in IA disease pathogenesis. Recent studies utilizing RNA sequencing analysis of whole synovial tissue biopsies, in addition to flow cell sorted immune and stromal cells reveal a complex transcriptional profile of the synovial tissue as a whole but also the transcriptional profile of specific synovial cells and the involvement of previously unappreciated molecular pathways (55–59). Synovial fibroblasts are the main invasive cells and key contributors in the pathogenesis of synovitis due to their capacity to produce pro-inflammatory cytokines, regulate the synovial invasion of immune cells and potentially regulate T cell activation and macrophage metabolism (60, 61). Importantly, a recent study identified different synovial fibroblast populations, based on the expression of FAPα and THY1, with distinct transcriptomic profiles and function, with the FAPα^+^THY1^−^ population being responsible for bone and cartilage degradation, while the FAPα^+^THY1^+^ population was associated with the more severe and persistent form of inflammatory arthritis (56). A similar approach allowed for the identification of two distinct macrophage sub-populations (MerTK^pos^TREM2^high^ and MerTK^pos^LYVE1^pos^), with the MerTK^pos^CD206^pos^ sub-population being associated with remission maintenance (55).

Several mechanisms including metabolic changes, hypoxia, cytokines, growth factors and immune-stromal cell crosstalk are involved in synovial inflammation; for instance, the hypoxic conditions of the synovial joint drives hypoxia-inducible factor 1-alpha (HIF-1α)-induced glycolysis in some of the mutual crosstalk between synovial fibroblast and immune cells, has been shown to evolve with the disease progression, suggesting that synovial fibroblasts have transitional properties in RA (59). Therefore, the joint is a very dynamic environment and a more in-depth characterization of immune and stromal cells at the site of inflammation, is required to elucidate disease pathogenesis and progression (1, 35, 54, 62, 63). Isolation of single cells suspension from the synovium by cell sorting, CyTOF, spatial transcriptomic and advanced imaging technology of synovial tissue will aid in improving our understanding of synovial cell crosstalk, activation, and disease progression.

In this review we present opportunities for novel functional characterization of synovial cells based on optimized flow cytometric analysis and downstream functional assays, including T cell activation and endocytosis. In addition, here we present advanced microscopic approaches in conjunction with RNAsequencing (RNAseq) as tools for a detailed analysis of synovial metabolism and cellular transcriptional changes. Importantly we describe key processes in sample preparation and discuss important considerations depending on downstream application of synovial cell suspensions. Thus, sample preparation is critical to any functional, imaging, metabolic or “omic” analysis.

## Generation of Synovial Single Cell Suspensions for Downstream Applications

**Figure 1:**
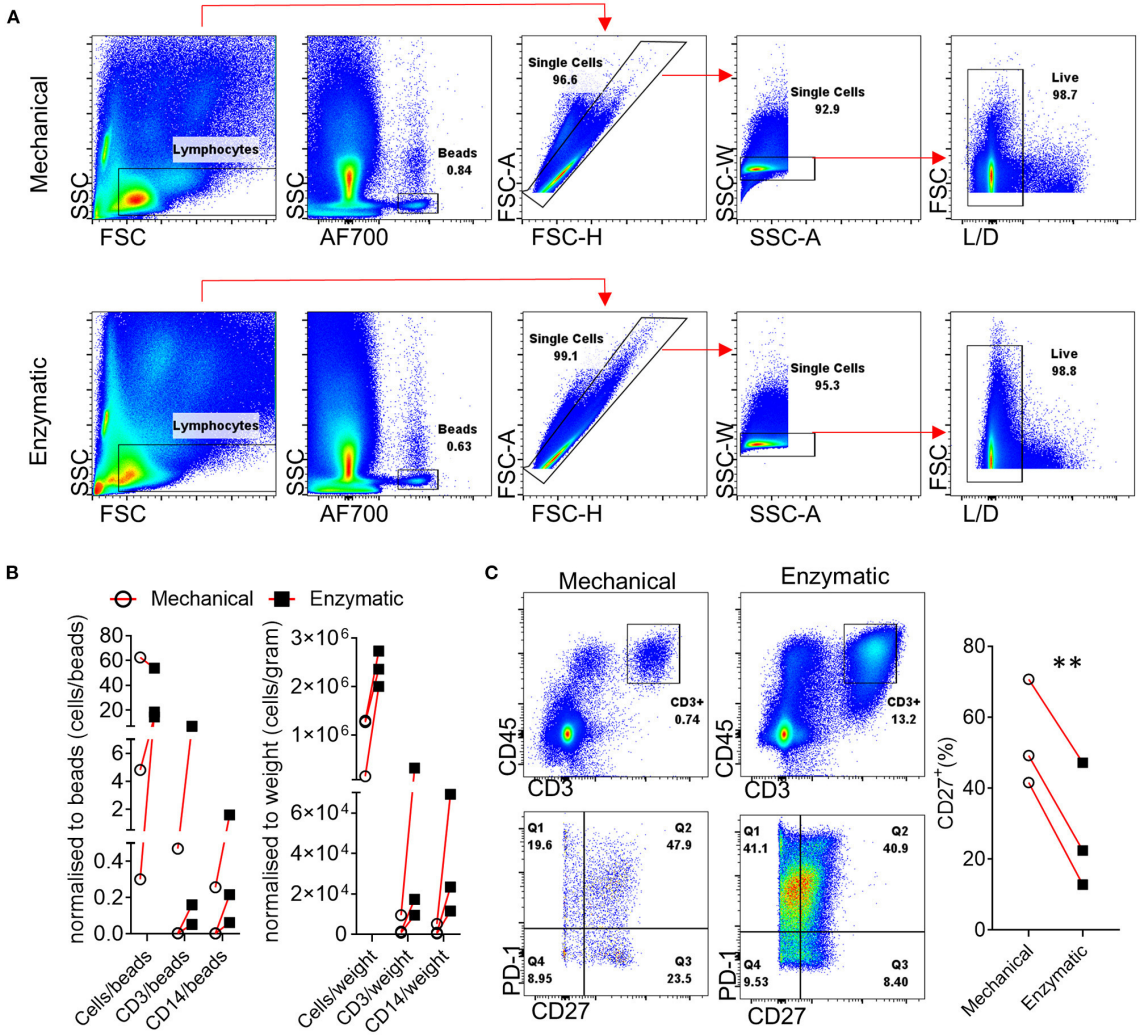
Effect of enzymatic digestion of synovial tissue biopsies on cell and marker recovery. **(A)** Representative flow cytometric analysis and gating strategy followed for paired enzymatic or mechanical synovial biopsy dissociation for the generation of a single cell suspension (*n* = 3). **(B)** Normalization of recovered, following enzymatic or mechanical digestion of paired synovial biopsies, CD3^+^ and CD14^+^ cells expressed as cells/counting beads or cells/weigh of synovial tissue. **(C)** Flow cytometric analysis of T cell CD27 expression following enzymatic or mechanical dissociation of paired synovial biopsies. Paired (same donor) synovial biopsies are shown, symbols indicate independent samples *n* = 3. Paired Students *T*-test was used for statistical analysis (**p = 0.003), *p*-values < 0.05 were considered significant.

One of the main functions of the synovial tissue is to regulate synovial fluid composition; as a result, the synovial tissue consists of fibrous, areolar tissues with a high collagen content (64). An important consideration is the method used for the dissociation of the synovial tissue and the generation of a single cell suspension that can then be utilized for flow cytometric, -omic, metabolic and functional analysis. To determine the best approach and achieve consistent cell recovery we tested combined enzymatic and mechanical dissociation to that of mechanical only dissociation of synovial tissue biopsies. Although both dissociation methods, led to a similar frequency of gated live cells (Figure 1A), when this was quantified and correlated to the ratio of cells/beads and cell/gram of biopsy, the digestion enzymes in combination with mechanical dissociation led to the release of considerably higher numbers of total live cells as well as CD3^+^ and CD14^+^ cells (Figure 1B), in agreement with previous observations by Donlin et al. (65). One important consideration when utilizing proteolytic enzymes that break down collagen fibers leading to the release of immune and stromal cells is the possibility of cleaving markers used for downstream analysis and consequently the generation of false negative results. We and others have previously highlighted this concern regarding the generation of synovial and intestinal cell suspensions (24, 66). Importantly, dissociation enzymes with collagenase activity belonging to different families of collagenases can have differential effects on masking expression of specific markers, including commonly used markers such as CD27, CXCR5, CD127, CD141, and CD4 creating further implications for the interpretation of results from different studies (24, 67). Indeed, we observed that T cell CD27 expression is significantly reduced following enzymatic dissociation compared to mechanical dissociation (^**^p = 0.003) (Figure 1C). Interestingly, we have previously shown that incubation of the synovial tissue B cells for 6 h post-digestion restored CD27 expression (24). Importantly, the masking effect of the dissociation enzymes can apply to a wide array of downstream analysis including conventional flow cytometric analysis, CyToF and even functional assays. Therefore, extensive optimization and characterization of staining panels and digestion protocols is required to confirm expression of surface markers on digested synovial tissues. Furthermore, periodic re-evaluation of staining panels if changes have been made to antibody clones and/or suppliers should also be performed.

## Cryopreservation of Synovial Tissue Biopsies and Single Cell Suspensions

**Figure 2:**
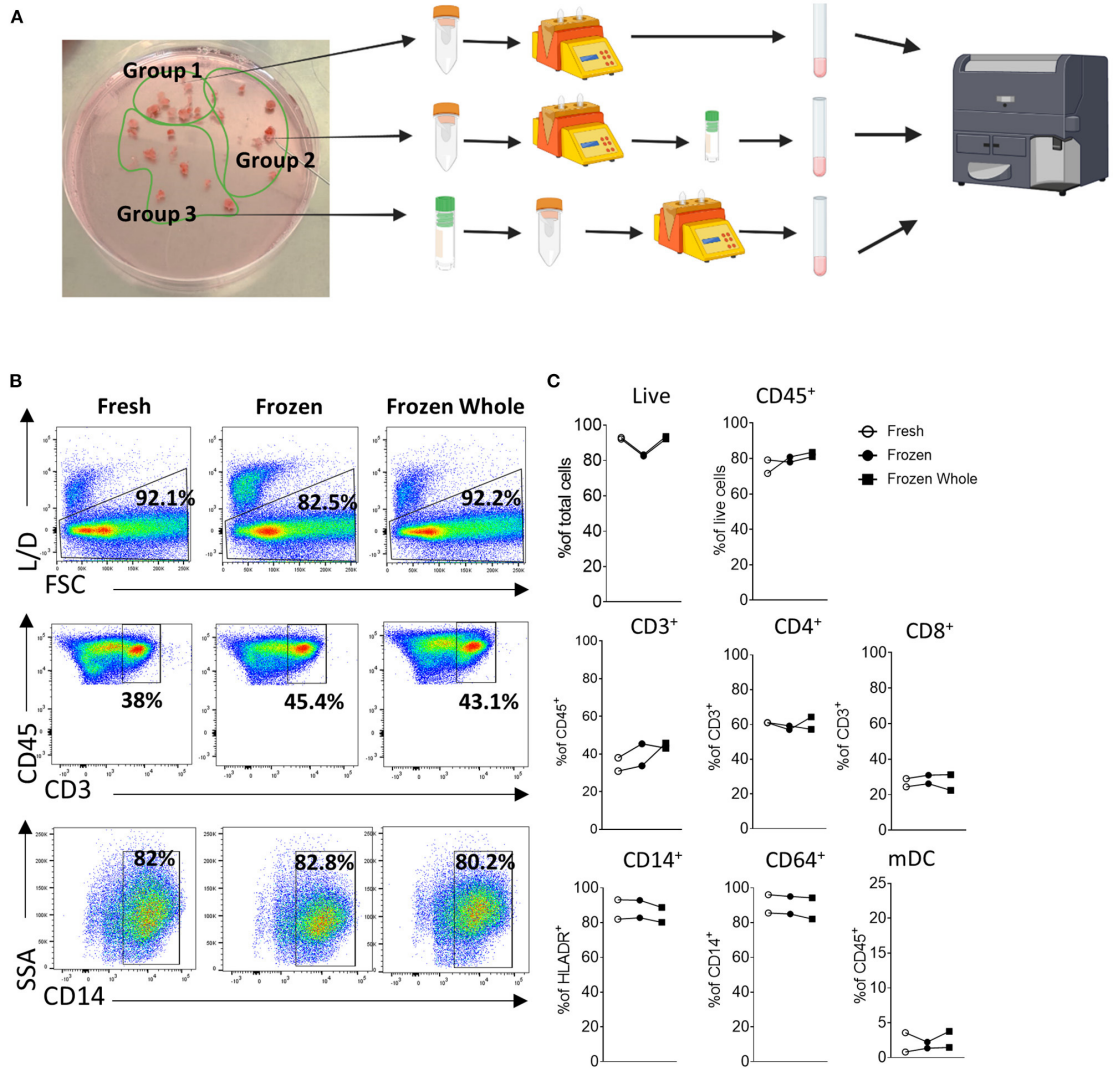
Cryopreservation of synovial tissue biopsies and single cell suspensions. **(A)** Schematic (Created with BioRender.com). Multiple biopsies from the same patients (*n* = 2) were randomly divided them in three groups bearing a similar number of biopsies. Group 1 and 2 synovial tissue biopsies were immediately digested with the enzymatic and mechanical protocol combination to establish a synovial cell suspension as described above. Group 1 (Fresh) was then stained for specific immune cell populations by flow cytometry immediately after digestion. Group 2 (Frozen) was digested and the resulting synovial cell suspension viably frozen in cryovial in a solution of FBS/DMSO 10%. Group 3- whole synovial tissue biopsies were instead viably frozen in FBS/DMSO 10%, prior to digestion and subsequently defrosted and digested. Group 2 and 3 were defrosted on the same day and stained for flow cytometry. **(B)** Representative dot-plot and **(C)** frequency of live cells, CD45^+^, CD3^+^ (as frequency of CD45^+^ cells), CD4^+^ and CD8^+^ (as frequency of CD3^+^ cells), CD14^+^ cells (as frequency of HLADR^+^ cells), CD68^+^ (as frequency of CD14^+^ cells) and mDC (as frequency of CD45^+^cells).

Donlin et al. (65), previously highlighted that the correct handling and cryopreservation/ thawing of synovial biopsies is essential for preserving cellular distribution, as shown by the similar viability and T/B and monocytes cells distribution in fresh vs. frozen synovial tissue. To further explore this, here we analyze three different methods for cellular analysis by flow cytometry, where we compared two different preservation methods to that of freshly digested synovial tissue from the same patient and stained cells by flow cytometry. For this purpose, we collected multiple biopsies from the same patients and randomly divided them in three groups bearing a similar number of biopsies (Schematic in Figure 2A). Group 1 and 2 synovial tissue biopsies were immediately digested with the enzymatic and mechanical protocol combination to establish a synovial cell suspension as described above (Figure 1). Group 1 (Fresh) was then stained for specific immune cell populations by flow cytometry immediately after digestion. Group 2 (Frozen) was digested and the resulting synovial cell suspension viably frozen in cryovial in a solution of FBS/DMSO 10%. Group 3- whole synovial tissue biopsies were instead viably frozen in FBS/DMSO 10%, prior to digestion and subsequently defrosted and digested (Figure 2A). Both group 2 and 3 were defrosted and stained for flow cytometry on the same day as described in methods (Supplementary File). The cellular suspensions obtained from all 3 groups were analyzed by flow cytometry for cell viability and frequency of immune cells. Interestingly, in all three conditions, similar viability was observed, with the frequency of live cells ranging between 82.9 and 92.9% and a percentage of CD45^+^ cells similar among the conditions (Figures 2B, C). Similarly, in-depth analysis of the cellular distribution displayed a similar frequency across the three groups for different immune cell types, including T cells: CD3^+^, CD4^+^, CD8^+^, monocytic cells: CD14^+^ monocytes, and CD64^+^ macrophage, as well as myeloid dendritic cells (mDC) (Figures 2B, C). Overall, these suggest that the correct cryopreservation of synovial biopsies does not alter the viability and cellular distribution of immune cells.

## Functional Studies Utilizing Synovial Tissue Cell Suspensions and Populations

**Figure 3:**
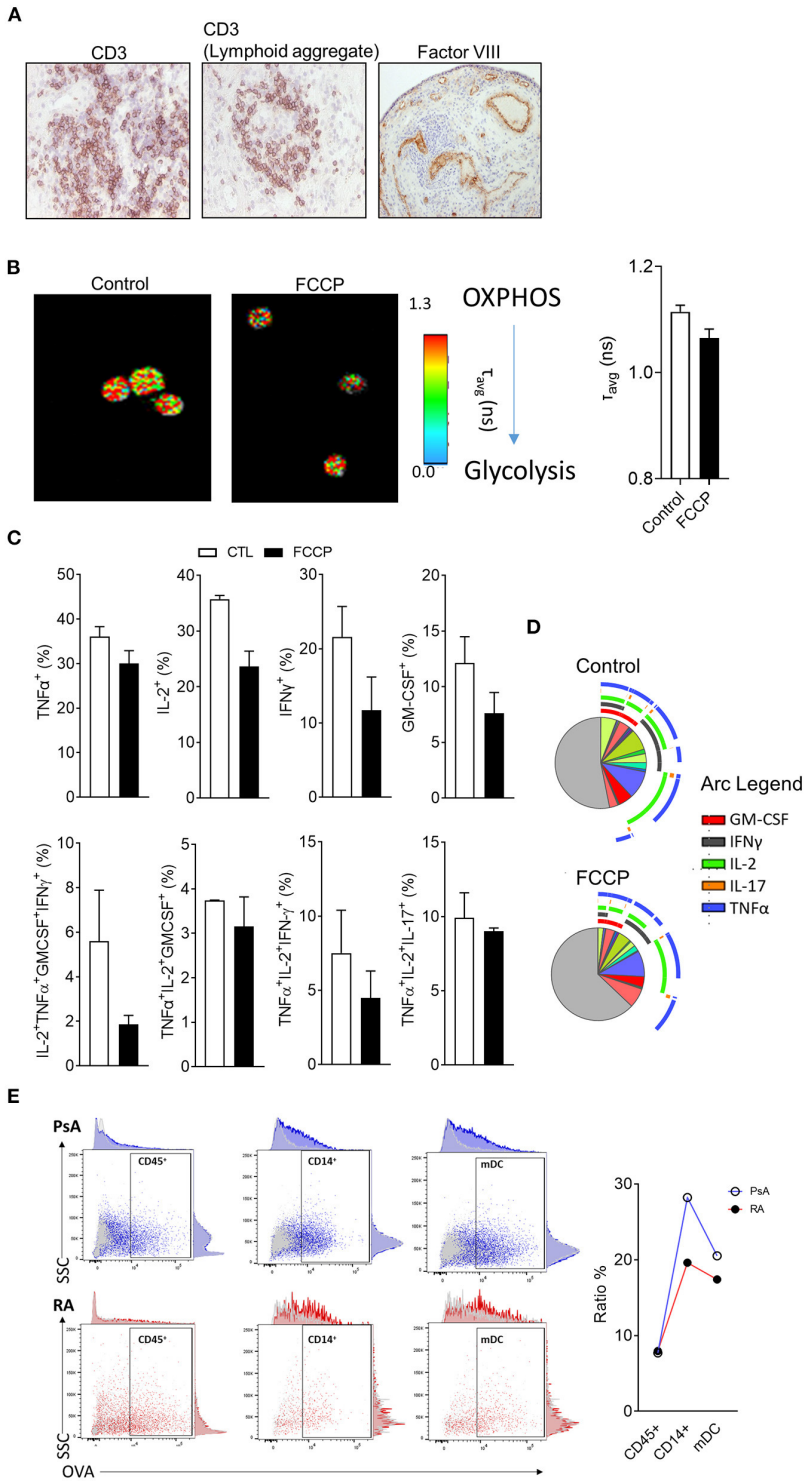
Metabolic and functional characterization of synovial tissue cells. **(A)** Representative H&E analysis of RA patient synovial tissue biopsies for expression of CD3 or Factor VIII. **(B)** Representative fluorescent lifetime imaging microscopy (FLIM) of synovial tissue cell suspension at baseline or following treatment with OxPhos inhibitor FCCP. **(C)** Flow cytometric analysis of synovial tissue T cell cytokine expression following stimulation *ex vivo* in the presence or absence of FCCP (*n* = 2). **(D)** SPICE algorithm analysis of T cell cytokine expression and polyfunctionality. Pie segments are indicative of percentage of CD4 T cells while pie arcs represent cytokine expression. **(E)** Endocytic activity assessment via flow cytometric analysis of fluorescent DQ-OVA uptake for the indicated synovial cell populations from synovial tissue biopsies of RA and PsA patients (*n* = 2)

As discussed above, histological evaluation, although providing limited phenotypical information of highly complex molecular mechanisms of synovial tissue, it displays a temporal snapshot of the cellularity of the synovial tissue, is highly accessible and can reveal immune and stromal cell organization linked to synovial pathotype, endotype and response (6, 27, 35, 68); Figure 3A shows representative images of RA synovial tissue CD3 T cell staining, where images show a diffuse infiltration pattern of CD3 T cells or a lymphoid aggregate pattern (Figure 3A). Previous studies have addressed the role of lymphoid aggregates in the synovium, highlighting their association with predictive clinical response (6, 36, 40). Lymphoid aggregates associate with specific cell signatures and disease progression, specifically, the T cell, B cell rich lympho-myeloid IA synovial pathotype shows enrichment of plasma cell signatures and enhanced disease activity (35). While the full extent of altered synovial immune processes resulting from aggregate formation remains to be elucidated, early studies show increased AID expression in support of synovial plasma cells and ACPA expression (36, 69). The lymphoid aggregate formations are now believed to be inversely correlated with IL-27 expression in the inflamed tissue, as well as with the expression of IL-17 and IL-21 at mRNA level (70, 71). The presence or absence of lymphoid aggregates can act as a predictor of response to treatment, as observed in pauci-immune patients, where the absence of B cell aggregate at baseline was correlated to be less inflammatory disease phenotype post-treatment (72). The increase in cellular infiltration observed in the IA synovium, is due to the formation of new blood vessels (as shown by Factor VIII staining- Figure 3A), which has been correlated with disease activity and response to therapy (2, 73). As discussed above, the angiogenesis formation pattern is a useful tool to discriminate between RA and PsA, with the latter forming distinct elongated, torturous blood vessels; in contrast, lining layer hyperplasia is more striking in RA than in PsA (18). This dysregulated synovial vascularisation coupled with increased cellular infiltration leads to reduced overall oxygen availability and results in the highly hypoxic microenvironment of the inflamed joint (1, 26, 70, 74, 75). Immune and stromal cells need to adapt to the hypoxic conditions, beyond adaptation, the increased availability of metabolic intermediates lactate, succinate and itaconate can enhance synovial inflammation (76–78). Increased lactate uptake by T cells can promote IL-17A and IFN- γ production while there is an increasing appreciation of metabolic reprograming involved in T cell polarization, effector function and retention at the inflamed tissue (79–81). Metabolic adaptation and nutrient sensing are also a characteristic of synovial stromal cells with fibroblast invasiveness regulated by mTOR and the amino acid transporters SLC7A5 and LAT1 132, 133 (82, 83). Importantly, immune-stromal cell interactions can potentially cause reciprocal metabolic changes leading to increased T cell activation and cytokine production and fibroblast responsiveness to proinflammatory signals (61).

Functional and metabolic characterization of synovial tissue cells can be challenging due to the relatively high number of cells required for analysis and accurate observation. Novel applications of fluorescent lifetime imaging microscopy (FLIM) can provide an assessment of metabolic profile on a single cell level without the need for labeling with external florescent probes (84). Two-photon FLIM (2P-FLIM), can be utilized in order to assess protein bound vs. free nicotinamide adenine nucleotide [NAD(P)H] and as a result provide an overview of the cell's metabolic state (85, 86). In the aforementioned application, FLIM is utilized to capture the fluorescence signal of NAD(P)H, a natural fluorochrome with importantly, distinct fluorescence lifetimes dependent on whether it is protein bound and therefore utilized in oxidative phosphorylation (OxPhos) (fluorescent lifetime of ~2.5 ns when bound compared to 0.4 ns if in free state) (87). Due to the single cell resolution that FLIM offers, it has previously been utilized for the characterization of the metabolic state of rare populations of cells (23, 24), herein, we have implemented FLIM in order to assess the metabolism of synovial tissue cell suspensions before or after treatment with OxPhos inhibitor carbonyl cyanide p-trifluoromethoxy-phenylhydrazone (FCCP) (Figure 3B) (88, 89). FCCP treatment results in decreased average times of fluorescent lifetime emissions indicative of a shift in the ratio between bound and free NAD(P)H in favor of free NAD(P)H and therefore, reduced OxPhos capacity of the cells.

**Figure 4:**
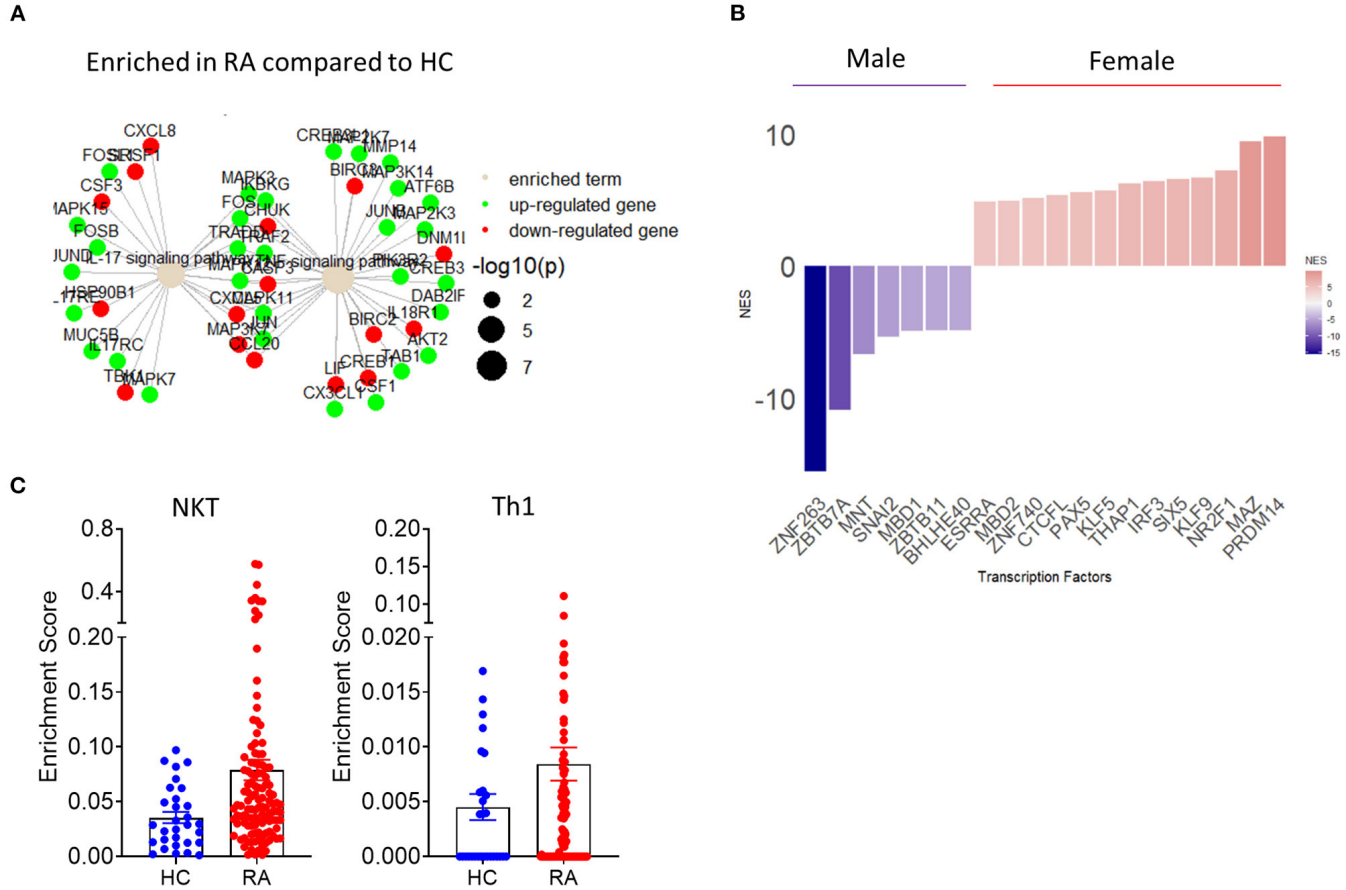
Implementation of transcriptomics in the study of synovial inflammation. **(A)** Term plot following pathway enrichment analysis of RA and HC synovial tissue biopsies. Only significantly upregulated (green) or downregulated (red) gene members of the pathway are shown. Gene dot size represents significance of change for the corresponding gene. **(B)** Transcription factor usage score of female compared to male RA patients based on differential expression of know target genes. **(C)** RA patient and HC synovial tissue enrichment scores for specific cell populations based on transcriptional signature deconvolution of bulk RNAseq data.

Previous studies have highlighted that fast response memory T cells and polyfunctional T cells characterized by the production of multiple cytokines, primarily rely on OxPhos instead of glycolysis in order to meet their energetic demands (23, 90). To investigate the functional consequence of OxPhos inhibition on synovial tissue T cells, directly *ex vivo* synovial tissue cell suspensions from IA patients were stimulated with PMA/Ionomycin in the presence/absence of FCCP (30 μM) followed by flow cytometric analysis for T cell derived TNF-α, IL-2, IFN-γ, IL-17A, and GM-CSF (Figure 4B). To examine cytokine co-expression and polyfunctionality, the supervised algorithm Simplified Presentation of Incredibly Complex Evaluations (SPICE, version 5.1) was used (91). SPICE is primarily a visualization algorithm based on Boolean gating of flow cytometric analysis data, the pie segments represent the relative frequency of CD4^+^ T cells and the arcs represent cytokine expression; overlapping arcs indicate simultaneous cytokine expression and therefore polyfunctionality (Figure 4C). Previous studies have highlighted correlation between synovial T cell polyfunctionality in RA and PsA with disease progression (23, 92). Importantly, highly pathogenic polyfunctional and resistant to autologous Treg mediated suppression, exTh17 and CD4^+^CD8^+^ T cells accumulate at the inflamed joint of RA patients (23, 93, 94). Synovial T cell polyfunctionality is not an epiphenomenon of synovial inflammation since it is detectable prior to clinical inflammation in “at risk” individuals (23). CD4^+^ T cells of synovial tissue biopsies incubated with FCPP showed decreased cytokine co-expression as a result of OxPhos inhibition and hence, polyfunctionality (Figures 4B, C). Specifically, we observed a decrease in the frequency of GM-CSF^+^, IFN-γ^+^, IL-2^+^ and TNF-α^+^ producing CD4^+^ T cells, as well as a decrease in specific cytokine expression combinations, including TNF-α^+^IL-2^+^IFNγ^+^GM-CSF^+^ and TNF-α^+^IL-2^+^IFNγ^+^ producing CD4^+^ T cells, paralleled by an increase in the frequency of cells not producing any of the cytokines analyzed (negative fraction) (Figures 3C, D). Metabolic adaptation is influenced by the local environmental conditions including oxygen and nutrient availability, additionally, metabolites have the potential to act as signaling molecules and alter the behavior of immune cells. Further characterization of *in situ* cell metabolism can be performed based on recent advances of mass spectrometry imaging (MSI) that enables the characterization of the proteomic and metabolomic profile of a cell and its neighbors on a sub-cell level (95). Importantly, MSI bypasses the need for the generation of a single cell suspension that may inadvertently impact cell metabolism.

Beyond the characterization of T cell derived cytokine production and metabolic profile, additional functional assays can be performed elucidating the characteristics of synovial tissue monocytes and dendritic cells (Figure 3E). Endocytosis mediated antigen uptake is a controlled process that reflects distinct developmental stages of dendritic cells (DC) with mature DC capable of performing antigen presentation, exhibiting reduced endocytic activity compared to immature DC (96). Importantly, endocytosis facilitates several immune functions including cytokine and cytokine receptor availability, signaling and metabolism for immune and stromal cells (97, 98). While the contribution of endocytosis in IA requires further study, we have recently showed that tofacitinib mediated JAK1/3 inhibition resulted in reduced endocytosis in patients with PsA, and at less extent RA, patient monocyte-derived DC (99). Importantly, optimized protocols allow for endocytic activity measurements to be performed on multiple populations simultaneously without the need for cell sorting from synovial tissue cell suspensions (see Supplementary File) (100). We observed similar endocytosis activity of the CD45^+^ population between PsA and RA patients (Figure 3E), calculated as % ratio between cells incubated at 37°C (specific uptake) and cells incubated at 4°C (non-specific uptake). However, when gating for more specific population within the CD45^+^ cells, we observed a decrease in endocytic activity in both CD14^+^ monocytes and mDC from RA patient when compared to PsA (Figure 3E), in agreement with previous observations (99). While identification and characterization of cell types and populations that associate with aspects of synovial pathology in arthritis, further characterization of the communication between synovial cell populations is needed. Altered distribution of synovial fibroblast subsets between distinct disease pathotypes characterized by differential immune cell composition, highlights that disease pathotypes are characterized by potentially unique immune-fibroblast cell interaction pathways (101). Further studies that incorporate scRNAseq and spatial transcriptomic analysis will elucidate cell-cell interactions and their connection to distinct arthritis pathotypes.

## RNA Sequencing in the Study of Synovial Inflammation

The recent implementation of bulk and single cell RNA sequencing has significantly impacted our understanding of molecular and transcriptomic pathway involvement in synovial inflammation. For instance, RNAseq of whole tissue biopsies from stratified patients based on active disease, low synovial inflammation or clinical remission has identified subclinical inflammation in RA patients in remission and a “core” gene expression signature associated with synovial inflammation (102). Extrapolation of synovial immune cell infiltration based on synovial tissue biopsy RNAseq analysis can be utilized to advice on therapeutic approaches, with lympho-myeloid RA pathotype patients requiring biological therapy at a higher frequency than diffuse-myeloid or pauci-immune RA (103). In addition to patient stratification or response to treatment, synovial tissue bulk RNAseq analysis can be utilized for the identification of specific mechanisms of synovial inflammation. For example, CD40 and CD40L expression and CD40-CD40L pathway members are significantly elevated in RA synovial tissue samples compared to healthy control (HC) subjects and associate with progression from arthralgia to early RA to established RA (58). Joint involvement in IA can follow different patterns with more frequent involvement of specific joints. Recent studies highlight transcriptional and epigenetic joint specific modulations of stromal cells, leading to distinct pro-inflammatory responses and invasive characteristics of synovial fibroblasts located in different joints (104, 105). This transcriptionally dictated positional memory of stromal cells can have a significant impact on the patient's response to therapy and exemplifies the need for more detailed characterization of the synovial microenvironment in IA (104, 105).

For the study of specific synovial cell populations directly *ex vivo* or following *in vitro* co-culture or activation, recent studies have utilized bulk and single cell RNAseq of flow cell sorted synovial cells (55, 106, 107). Single cell RNAseq analysis of flow cell sorted synovia CD4 and CD8 T cells revealed enrichment in activated memory CD8 T cells in PsA patient synovial fluid and importantly, convergence of T cell receptor gene signatures, highlighting shared CD8 T cell clone involvement across PsA patients (106). In addition to whole synovial tissue analysis, scRNAseq approaches can be used for specific applications on synovial cell microcultures (55). Recent scRNAseq studies of synovial fibroblasts cocultured with MerTK^−^CD206^−^ or MerTK^+^CD206^+^ synovial macrophages shows that MerTK^−^CD206^−^ macrophages evoke proinflammatory transcriptional adaptation of synovial fibroblasts (55).

Importantly, fast developing bulk and scRNAseq analysis can be combined in novel approaches for the identification of biomarkers of disease and response to treatment. RNAseq analysis provides a temporal snapshot of the joint, the standardization of those procedures and the growing ability to perform minimally invasive repeat ultrasound biopsy or key hole arthroscopic biopsy can mitigate this limitation of RNAseq and guide the investigation of novel biomarkers and pathways of inflammation (108). The aforementioned approach is further supported by novel studies that have identified pathogenic as well as protective roles of TNF depending on its cellular source and temporal expression (109). Importantly, the utilization of sequential biopsies in combination with high dimensionality transcriptomic analysis show superior efficacy in patient stratification compared to histological approaches and may reveal why certain patients show inadequate response to treatment, therefore leading to improved patient stratification and clinical practice (110). Recent combinatorial use of bulk and single cell RNAseq revealed the presence of three major fibroblast clusters with distinct transcriptomic profiles based on expression of CD34 and THY1(57). CD34^+^ fibroblasts are characterized by expression of proinflammatory cytokines including IL-6, while CD34^−^THY1^−^ and CD34^−^THY1^+^ fibroblasts promote osteoclastogenesis and may contribute to bone erosion (57). Interestingly, subsequent histological analysis of RA patient synovial tissue samples based on the markers identified by RNA sequencing, showed distinct synovial localization of the three identified major fibroblast clusters (57). Studies in synovial tissue organization, cell-cell interactions and transcriptomic profiles will become more elaborate with the expected emergence of spatial transcriptomics (111). However, currently publicly available RNAseq data can be repurposed and reanalyzed to answer further questions regarding synovial pathogenesis. The accelerating introduction of novel algorithms of differential gene expression and pathway enrichment can lead to improved visualization of molecular pathways involved in IA pathogenesis (Figure 4A) (112). Importantly, as resolution of transcriptional regulation increases and transcription factor regulation of downstream target genes is identified, transcription factor activity can be inferred bioinformatically based on differential gene expression data (Figure 4B). Preliminary analysis utilizing the aforementioned approach shows a clear demarcation of female and male RA patients based on synovial tissue transcription factor activity with female patients showing increased activity of the epigenetic regulator PRDM14 (58, 113–115) (Figure 4B). One of the main disadvantages of whole synovial tissue RNAseq is the inability to separate between different cell types and assess tissue compositions and cell specific transcriptional signatures. New analysis approaches help provide an overview of whole tissue cellular composition by deconvolution of bulk RNAseq data based on predetermined transcriptional signatures associated with specific cell populations (116). Such approaches can provide an overview of the cellularity of the synovial landscape utilizing bulk whole tissue RNAseq data and allow for comparison of cell specific enrichment scores between sample groups. As shown in Figure 4C, there is an increase in NKT and Th1 associated gene signatures in RA patients compared to HC synovial tissue.

The implementation of spatial transcriptomics and novel scRNAseq and bulk RNAseq analysis approaches will revolutionize our understanding of synovial inflammation. Importantly, advances in microfluidic applications for RNAseq and streamlined analysis workflows, will reduce the costs associated with clinical application of transcriptomic analysis and their utilization in personalized medicine (117). However, -omic approaches provide a temporal or spatio-temporal view of the inflamed joint, therefore functional assay involvement will need to be more frequently and robustly implemented to match advances in RNAseq. The exciting and novel applications of high throughput techniques and functional studies need to be based on well-optimized synovial tissue handling and dissociation approaches.

## Conclusions

**Figure 5:**
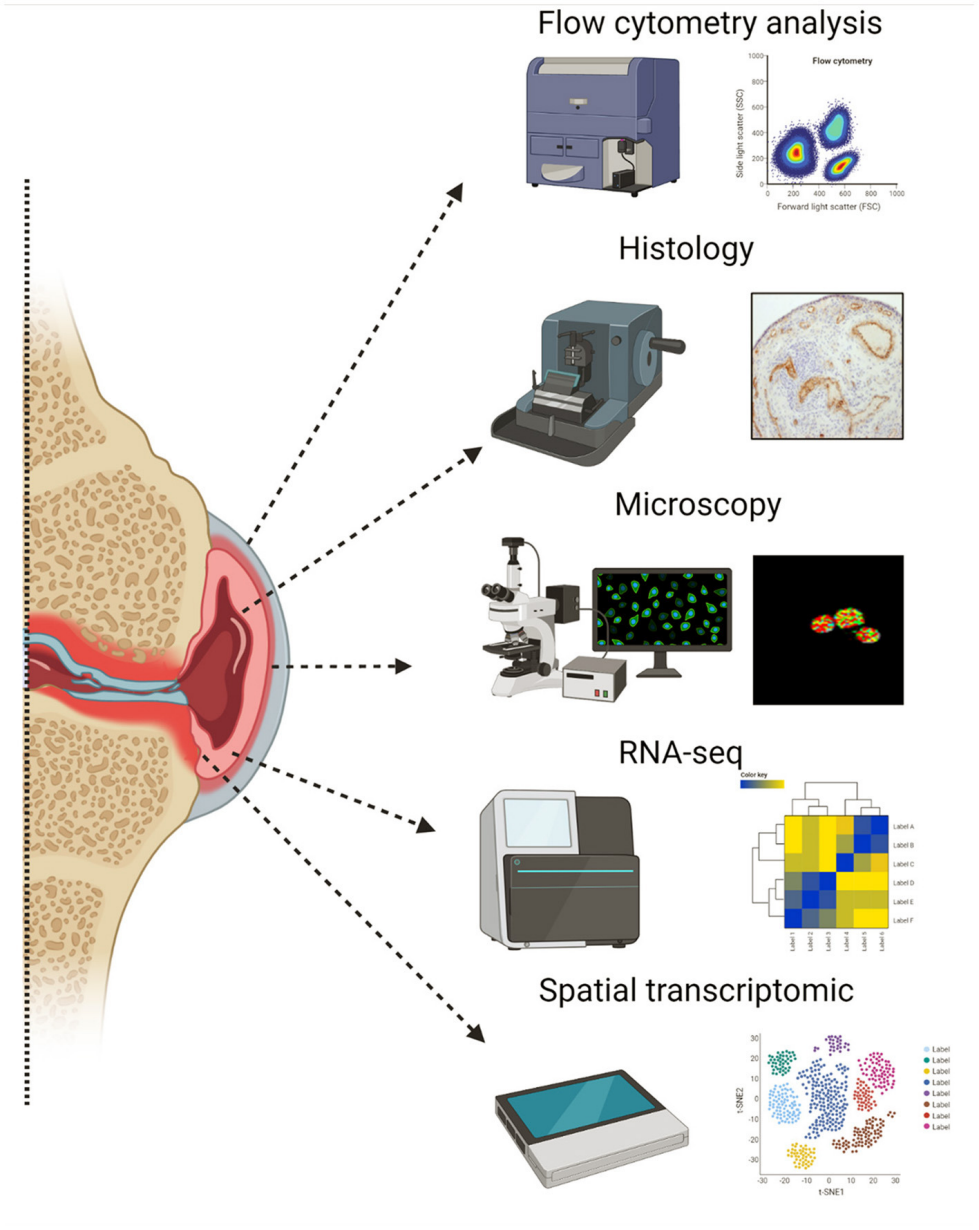
Illustration of key techniques used in synovial tissue research. Created with BioRender.com.

Increased synovial tissue availability and synovial tissue research have been instrumental in recent advances in our understanding of the immunological mechanisms involved in joint inflammation and have subsequently led to improved clinical practice. Herein, we outline important steps and considerations for the successful analysis of synovial tissue biopsies. Additionally, we describe opportunities for the utilization of –omic approaches and novel functional assay applications in the study of synovial immune and stromal cell responses (Figure 5).

The interrogation of synovial tissue cells and immune mechanisms contributing to synovial pathology, depends on synovial tissue processing and cryopreservation. Importantly, the compatibility of tissue dissociation method and downstream application needs to be examined depending on the relevant experimental approach and hypothesis (66). Another point of consideration is the need and method of synovial tissue cryopreservation. Cryopreservation can potentially help alleviate aspects of batch-to-batch variation and allow for direct comparison of multiple samples from distinct patient groups or time points. However, it needs to be noted that downstream functional or transcriptomic characterization of synovial tissue samples may be impacted by cryopreservation and testing of specific cryopreservation methods and adaptation of cell sorting requirements based on downstream applications is advised (118).

High dimensionality flow cytometric and RNAseq analysis provide a temporal snapshot of the synovial tissue, therefore, to appreciate the significance of identified molecular pathways, functional assays are essential. A key obstacle in synovial tissue research is the low number of available cells; the implementation of novel approaches, optimized protocols and advanced microscopy-based techniques can provide opportunities for functional interrogation of synovial cells bypassing the need for high numbers of available cells. One exception to the high cell number requirement for the implementation of functional studies, is synovial fibroblasts due to available, well-characterized and optimized protocols of *in vitro* expansion of these cells. As a result fibroblasts from IA patient synovial biopsies can be utilized in invasion and migration assays following response treatment, metabolic characterization and even co-culture with immune and stromal cells, including the recently characterized pro-inflammatory MerkTK^−^CD206^−^ synovial tissue macrophages (17, 55, 61, 119). These studies have significantly improved our understanding of synovial fibroblast involvement in IA pathogenesis, however, once removed from the synovial environment and expanded *in vitro* transcriptional characteristics of specific synovial fibroblasts are potentially altered (120). Therefore, investigation of the full potential and involvement of synovial fibroblasts in IA pathogenesis may require novel experimental approaches. The inflamed joint is a dynamic environment of constant adaptation of immune and stromal cells to unique environmental conditions of oxygen and nutrient availability (121). Metabolic processes are fundamental in regulating inflammation, as such metabolism is not a steady state, rather a continuous adaptation to changing environmental conditions (122). Metabolic states can change rapidly due to altered nutrient, metabolite and oxygen availability, with recent reports demonstrating that metabolism can be significantly impacted during the process of flow cell sorting (123). Techniques that allow for examination of cell metabolism following minimal handling can potentially provide a more accurate and representative assessment of metabolic processes at the site of inflammation. Early studies of histological examination of synovial tissue biopsies demonstrated a fundamental role for T cells in IA pathogenesis (6). Recent advances in high dimensionality flow cytometric data analysis, exploration and visualization, have substantiated histological findings of T cell involvement leading to growing appreciation of T cell polyfunctionality in IA pathogenesis (23, 92, 93). With the expanding utilization of single cell RNAseq in the study of synovial tissue inflammation, further development and implementation of functional assays will be important for the translatability of transcriptomic approaches.

Recent applications of single cell RNAseq and mass cytometry have considerably increased the resolution of the synovial landscape due to the identification of transcriptionally distinct synovial and immune cell clusters (55, 56, 106, 120, 124). While scRNAseq approaches have distinct advantages over bulk RNAseq analysis, the rapid and continues improvement of analysis algorithms for bulk RNAseq datasets can lead to their re-utilization for further interrogation of synovial inflammation pathogenesis and patients' stratification (58, 125). Novel approaches for the interrogation of cell-cell interactions based on transcriptomic data are required and can lead to significant advances in the development of novel targeted therapeutic interventions that will disrupt specific cell-cell interactions. Such tools have been described recently, however, their application in the study of complex systems will need extensive testing and validation (126). An important milestone in synovial tissue research that will unlock several opportunities for novel targeted therapeutic approaches will be the interrogation of cell-cell interactions using transcriptomic data in conjunction with “geographical” knowledge of immune and stromal cells based on recent advances in spatial transcriptomics (127). Implementation of spatial transcriptomics will validate recent attempts to transcriptionally characterize the synovial cell landscape based on scRNAseq analysis.

Synovial tissue research is undergoing an exciting transformation with the implementation of –omic approaches and novel mechanistic, functional interrogations of synovial tissue pathogenesis; these opportunities can exacerbate known obstacles and present new ones. Overcoming those has the potential to propel forward our understanding of IA pathogenesis and lead to substantial advances in clinical practice.

## Author Contributions

AF, UF, and VM: conceptualization, visualization, and supervision. AF, VM, ZE, AG, NN, UF, and MM: methodology and writing—original draft preparation. AF and VM: software. AF, VM, NN, ZE, and UF: validation, formal analysis, and investigation. UF and VM: resources and funding acquisition. AF, VM, UF, NN, AG, and ZE: data curation. UF: project administration. All authors contributed to the article and approved the submitted version.

## Funding

This research was funded by Science Foundation Ireland 20/FFP-P/8666 and 21/PATH-S/9327, Center for Arthritis and Rheumatic Diseases, CARD-2019-01, and Arthritis Ireland.

## Conflict of Interest

The authors declare that the research was conducted in the absence of any commercial or financial relationships that could be construed as a potential conflict of interest.

## Publisher's Note

All claims expressed in this article are solely those of the authors and do not necessarily represent those of their affiliated organizations, or those of the publisher, the editors and the reviewers. Any product that may be evaluated in this article, or claim that may be made by its manufacturer, is not guaranteed or endorsed by the publisher.
