# Supplementary material for: Inside the Joint of Inflammatory Arthritis Patients: Handling and Processing of Synovial Tissue Biopsies for High Throughput Analysis
Source: Front Med (Lausanne). 2022 Mar 14;9:830998. doi: 10.3389/fmed.2022.830998 (PMC8967180; doi:10.3389/fmed.2022.830998)
Supplement: Supplementary file 1 [file Data_Sheet_1.docx]

**Methods**

**List of abbreviations**

IA Inflammatory Arthritis

RA Rheumatoid Arthritis

PsA Psoriatic Arthritis

RMD Rheumatic and Musculoskeletal Diseases

bDMARD Biologic disease-modifying antirheumatic drug

FLS Fibroblast-like synoviocyte

ACPA Anti-citrullinated protein antibodies

ECM Explant conditioned media

HIF-1α Hypoxia-inducible factor 1-alpha

DC Dendritic cells

HC Healthy Control

RNAseq RNAsequencing

FLIM Fluorescent Lifetime Imaging Microscopy

2P-FLIM Two-photon FLIM

OxPhos Oxidative Phosphorylation

FCCP Carbonyl cyanide p-trifluoromethoxy-phenylhydrazone

SPICE Simplified Presentation of Incredibly Complex Evaluations

**Patient Recruitment and sample collection.**

Synovial tissues biopsies were obtained at arthroscopy from the Rheumatology Department of St. Vincent’s University Hospital (Dublin, Ireland). Ethics approval for this study was performed by the St. Vincent's University Hospital and Medical Research Committees and in accordance with the Declaration of Helsinki (IRB RS18-055).

**Digestion of synovial tissue biopsies.**

Synovial tissue biopsies obtained at the time of arthroscopy were digested either mechanically, or with a combination of mechanical and enzymatic digestion, to yield a cell suspension of synovial tissue cells. GentleMacs dissociator alone (mechanic digestion only) or in combination with and the human tumour dissociation kit (mechanical/enzymatic digestion) (Milteniy Biotech, Germany) were used, according to manufacturer’s instructions. Briefly, following arthroscopy, synovial tissue was sectioned into small pieces and divided in two groups, a digestion enzyme mix composed of 4.7 mL serum-free and antibiotic-free RPMI medium supplemented with 200μL of enzyme H, 100μL of enzyme R and 25μL of enzyme A were added to the gentleMACS C tube for the mechanical/enzymatic digestion only. Using the GentleMACS program; m_spleen_4, mechanical stress was applied to the synovial tissue for 60 sec. Samples were then incubated at 37⁰C for 30 min under constant rotation using the MACSmix Tube Rotator (Miltenyi Biotech). The samples were subsequently exposed to a second mechanical agitation using the m_brain_03 gentleMACS program and incubated for a further 30 min at 37⁰C. A final mechanical agitation was applied for 30 sec using the gentleMACS h_tumor_03 program. The resulting cell suspension was passed through a 70 μm cell strainer (1).

**Cryopreservation and thawing of synovial tissue biopsies.**

Synovial tissue biopsies were digested with the combination of mechanical/enzymatic digestion as described above. Biopsies were either digested freshly (group 1 and 2) or were firstly cryopreserved whole (group 3) and then subsequently defrosted and digested. Cryopreservation of either the digested cell suspension or the whole biopsies were obtained by quickly re-suspending them in a freezing solution of 10% DMSO in FBS. Cryovials were then placed in an insulated Mr. Frosty™ Freezing Container (Thermofisher) and placed at -80°C for at least 2 days. The digested cell suspensions were thawed rapidly by warming the cryovial in a 37°C water bath and the suspension was then washed in pre-warmed media to remove the DMSO. The whole biopsies were thawed rapidly by warming the cryovial in a 37°C water and collected in a petri dish with warm media. They were then washed in PBS before dissociating them as described above. Cells were then stained for flow cytometric analysis.

**Flow cytometric analysis**

For the flow cytometric analysis of cellular distribution between fresh vs frozen, synovial tissue cell suspension were collected and washed in ice cold PBS prior to incubation with LIVE/DEAD™ Fixable Near-IR dye (BioSciences). An Fc receptor blocking step was performed by incubating the cells with TruStain FcX Fc blocking solution (Biolegend). Cells were then stained for 30 min at 4°C with the following antibodies: CD45 FITC (Biolegend, clone H130), CD64 PerCP-Cy5.5 (BioScience, clone 10.1), CD4 Alexa Fluor® 700 (Biolegend, clone A161A1), HLA-DR Brilliant Violet 521 (Biolegend, clone G46-6), CD3 Brilliant Violet 510 (Biolegend, clone OKT3), CD11c Brilliant Violet 650 (Biolegend, clone Bu15), CD14 PE (Biolegend, clone M5E2), CD19 PE/Dazzle (Biolegend, clone HIB19), CD8 PE/Cyanine5 (Biolegend, clone HIT8a). Samples were acquired using the Fortessa LSR II Flow Cytometer (BD) and analysed using Flowjo software v10.7 (Treestar Inc.).

**Cell stimulation and intracellular cytokine staining**

For biopsy cell suspension stimulation and cytokine detection, 3x10^5^ cells/ well were placed in a flat bottom 96-well plate in 100μl of cRPMI (RPMI GLUTAMAX, +10% FBS, + Penicillin/Streptomycin) (ThermoFisherScientific). Cells were then incubated for 3 hours with 30µM FCPP or vehicle (Control). Subsequently, cells were stimulated with cell stimulation cocktail (PMA/Ionomycin) (eBiosciences) for one hour prior to the addition of 1X Brefeldin-A and 1X Monensin (both from ThermoFisher) for a further four hour incubation. Cells were first stained for surface markers (CD3 and CD4) and subsequently stained for intracellular markers (GM-CSF, IFNγ, IL-2, TNF and IL-17) using the eBioscience™ Foxp3/Transcription Factor Staining Buffer Set (Lifesciecnces), as per the manufacturer’s protocol.

**Endocytic activity**

Endocytic activity was evaluated by flow cytometry analysis as previously described (2, 3). Synovial tissue biopsies were isolated and digested (as above) and washed in phosphate-buffered saline (PBS). The resulting single cell suspension was re-suspended in complete medium and transferred to flow-cytometry tubes containing 4 µl DQ^TM^ Ovalbumin (DQ OVA, Molecular Probes). Tubes were incubated in parallel at 4 and 37 ºC for 15 mins and washed twice in cold FACS buffer. Cells were subsequently blocked with a human FcγR-binding inhibitor prior to staining with specific antibodies CD45 PE/CY5 (Clone HI30), CD11c PerCP/Cy5.5 (clone [Bu15](https://www.biolegend.com/en-us/search-results?Clone=Bu15)), HLA-DR Brilliant Violet 421 (Clone G46-6), CD14 Brilliant Violet 510 (Clone  M5E2), as described above. The incorporated fluorescence of the fluorescent reporters DQ-OVA (receptor-mediated endocytosis) was analysed by flow cytometry in the CD209/CD14^+^ gated cells, by following the excitation with the 488 nm laser and fluorescence using the 530/30 bandpass filter. The frequency of cells incorporating the DQ-OVA was calculated by subtracting cells incubated at 37 °C (specific uptake) with cells incubated at 4 °C (non-specific uptake).

**RNAseq**

Analysis was performed on previously obtained RNAseq data of RA (n=118), and HC (n=44) synovial tissue biopsies (7). Briefly, quality of RNA was evaluated using an Agilent bioanalyzer followed by RNAseq by Q2 Solutions (Morrisville, NC). Sequencing libraries were prepared on Truseq stranded total RNA using the Illumina Ribo- Zero protocol. Sequencing of pooled libraries was performed on an Illumina HiSeq 2000, and raw read quality was evaluated using FastQC. Raw reads were trimmed based on sequence quality and adaptors leading to an average number of clusters per sample of 8.9×10^7^. Reads were then aligned to the human reference genome b37.3 using STAR V.2.4.15. Quantification of aligned reads was performed using RSEM V.1.2.14 with the University of California Santa Cruz transcriptome model (accessed on 17 March 2014) that included lincRNAs from Ensembl V.75. Aligned data were subjected to evaluation of quality using several metrics including mapping rate, coverage and deviation from principal component analysis (PCA). Differential gene expression analysis was performed in R with package *DESeq2* (v1.32), genes with less than 10 counts were filtered out, followed by pathway enrichment analysis with package *pathfindR* (v1.6.3) (4, 5). Transcription factor usage estimation was performed with *dorothea* (v1.4.1) (6). Enrichment scores for specific cell populations was performed based on transcriptional signature identification and deconvolution of the RA patient and HC synovial tissue biopsy bulk RNAseq analysis data with package xCell (v1.1) (7).

**Histology**

Synovial biopsies obtained at arthroscopy were snap frozen in OCT compound and stored at −80 °C. Seven-micron-thick sections were cut using a cryostat and placed on glass slides coated with 2 % 3-amino-propyl-triethoxy-silane (Sigma-Aldrich Ireland Ltd, Dublin, Ireland), dried overnight at room temperature and stored at −80 °C. Tissue sections were fixed in acetone for 10 min and air-dried. Non-specific binding was blocked using 10 % casein buffer. A routine three-stage immunoperoxidase labelling technique incorporating avidin-biotin-immunoperoxidase complex (Dako, Glostrup, Denmark) was used. Sections were incubated with primary mouse monoclonal anti-CD3 and Factor VIII (DAKO, Glostrup, Denmark) at room temperature for 1 hour. Colour was developed in solution containing diaminobenzadine-tetrahydrochloride (Sigma-Aldrich), 0.5 % H_2_O_2_ in phosphate-buffered saline (PBS) buffer (pH 7.6). Slides were counterstained with haematoxylin (BDH Laboratory Supplies, Poole, UK) and hydration and fixation was performed through a series of IMS and xylene solutions. Sections were mounted using DPX mountant (BDH Laboratory Supplies). Images were captured using Olympus DP50 light microscope and analysis software (Soft Imaging System Corporation).

**Two-photon fluorescence lifetime imaging microscopy**

In order to investigate the metabolic state of synovial biopsies, two-photon fluorescence lifetime imaging microscopy (2P-FLIM) was used. This technique relies on endogenous fluorophores such as nicotinamide adenine nucleotide (NAD(P)H) to obtain an image and infer on the cellular metabolic state (8). Its potential is correlated with the ability to distinguish between protein-bound and free NAD(P)H due to NAD(P)H self-quenching process. This results in a longer fluorescence lifetime of ~2.5 ns for protein-bound NAD(P)H and a short lifetime of ~0.4 ns for free NAD(P)H. It has been shown that an increase in the fraction of protein-bound NAD(P)H results from an increase in oxidative phosphorylation (OxPhos) metabolic dependence (6,9,10). Synovial biopsies were immediately transferred to a 18-well 15-µ-Slide (Ibidi) and treated with 30µM FCPP or vehicle (Control) for 1h. 2P-FLIM was performed using an upright Olympus BX61W1 multiphoton microscopy system equipped with a Titanium: sapphire laser (Chameleon Ultra, Coherent), a water-immersion objective (25× Olympus 1.05NA) and a temperature-controlled stage (37°C). NAD(P)H excitation was performed at a wavelength of 760 nm and fluorescence emission was isolated with a 455/90 nm bandpass filter. Fluorescence lifetime decay measurements were obtained using a PicoHarp 300 TCSPC system operating in the time-tagged mode coupled with a PMA hybrid detector (PicoQuanT GmbH, Germany) at 256 time bins per pixel, at least three images were acquired for each sample. The overall decay curves were generated and fitted using a two-component fitting to differentiate between the proteinbound (τ1 ) and free (τ2 ) NAD(P)H (Eq.1): I ( t ) = α1e − t τ1 + α2e − t τ2 + c(1) I(t) corresponds to the fluorescence intensity measured at time t after laser excitation; α1 and α2 represent the fraction of the overall signal comprise of a long and short lifetime components, respectively; C corresponds to background light. To easier understand the change of both fluorescence lifetime components, we calculated the average lifetime (τavg) of NAD(P)H (Eq.1).

tavg = [( α1×τ1 ) +( α2×τ2 )]/( α1+α2 )

**References**

1. McGarry T, Hanlon MM, Marzaioli V, Cunningham CC, Krishna V, Murray K, et al. Rheumatoid arthritis CD14(+) monocytes display metabolic and inflammatory dysfunction, a phenotype that precedes clinical manifestation of disease. *Clin Transl Immunology.* 2021;10(1):e1237.

2. Canavan M, Marzaioli V, Bhargava V, Nagpal S, Gallagher P, Hurson C, et al. Functionally Mature CD1c(+) Dendritic Cells Preferentially Accumulate in the Inflammatory Arthritis Synovium. *Front Immunol.* 2021;12:745226.

3. Marzaioli V, Canavan M, Floudas A, Wade SC, Low C, Veale DJ, et al. Monocyte-Derived Dendritic Cell Differentiation in Inflammatory Arthritis Is Regulated by the JAK/STAT Axis via NADPH Oxidase Regulation. *Front Immunol.* 2020;11:1406.

4. Ulgen E, Ozisik O, and Sezerman OU. pathfindR: An R Package for Comprehensive Identification of Enriched Pathways in Omics Data Through Active Subnetworks. *Front Genet.* 2019;10:858.

5. Love MI, Huber W, and Anders S. Moderated estimation of fold change and dispersion for RNA-seq data with DESeq2. *Genome Biol.* 2014;15(12):550.

6. Garcia-Alonso L, Iorio F, Matchan A, Fonseca N, Jaaks P, Peat G, et al. Transcription Factor Activities Enhance Markers of Drug Sensitivity in Cancer. *Cancer Res.* 2018;78(3):769-80.

7. Aran D, Hu Z, and Butte AJ. xCell: digitally portraying the tissue cellular heterogeneity landscape. *Genome Biol.* 2017;18(1):220.
